# Enhancement of the Storage Potential of Farmed Rainbow Trout (*Oncorhynchus mykiss*) by Using Algal (*Cystoseira myrica* and *Cystoseira trinodis*) Extract–Ice Combinations

**DOI:** 10.3390/foods14030371

**Published:** 2025-01-23

**Authors:** Shima Ahmadi, Parastoo Pourashouri, Bahareh Shabanpour, Santiago P. Aubourg

**Affiliations:** 1 Department of Seafood Processing, Faculty of Fisheries and Environmental Sciences, Gorgan University of Agricultural Sciences and Natural Resources, Shahid Beheshti St., Gorgan 4913815739, Golestan, Iran; sh.ahmadi171@yahoo.com (S.A.); pourashouri.p@gau.ac.ir (P.P.); shabanpour@gau.ac.ir (B.S.); 2 Department of Food Technology, Marine Research Institute (CSIC), c/Eduardo Cabello 6, 36208 Vigo, Spain

**Keywords:** *Cystoseira* sp., extracts, farmed rainbow trout, chilling, ice medium, microbial development, lipid damage, quality, polyphenol content

## Abstract

An attempt to apply extracts of the brown algae *Cystoseira myrica* and *Cystoseira trinodis* for the quality enhancement of fish was carried out. Aqueous, ethanolic, and aqueous–ethanolic (1:1, *v*/*v*) extracts of both algae were included, respectively, in the icing system employed for the chilled storage of farmed rainbow trout (*Oncorhynchus mykiss*). Chemical and microbiological quality indices were determined for a 0–16-day storage period. At the end of the experiment, all alga-treated fish revealed lower (*p* < 0.05) pH values and lower (*p* < 0.05) lipid hydrolysis (free fatty acid assessment) and oxidation (thiobarbituric acid index) development when compared to Control samples. Regarding microbial activity development (aerobe, psychrophilic, *Enterobacteriaceae*, proteolytic, and lipolytic counts), lower average values were detected in most cases in fish corresponding to alga-treated batches; preservative effects were found more important at advanced storage times. In general, water and water–ethanol extracts led to higher (*p* < 0.05) inhibitory effects than their counterpart ethanol extracts. Higher (*p* < 0.05) total polyphenol values were detected in water and water–ethanol extracts of both algae than in their counterpart extracts obtained only with ethanol. A novel, simple, and practical strategy for the quality enhancement and commercialization of chilled farmed rainbow trout is proposed by employing different extracts obtained from both *Cystoseira* species.

## 1. Introduction

Rainbow trout (*Oncorhynchus mykiss*) is a highly valuable species for aquaculture due to its ability to be raised and high growing capabilities [1,2]. With a production volume of approximately 740,000 tons in 2020, it is considered the most widely farmed salmonid species in freshwater [3]. Currently, this species is farmed all over the world and shows the ability to live within a wide range of water temperatures, i.e., from 0 to 23 °C. According to its high nutritional quality, rainbow trout farming can contribute to addressing the increasing demand for farmed fish production to help feed the ever-growing human population [2,4]. However, the shelf-life time of this species is short due to being highly susceptible to chemical deterioration and microbial spoilage. Although several preservative strategies (packaging and the addition of essential oils or plant extracts) have been successfully applied for maintaining the quality of rainbow trout as a fresh product [5,6], further research is still needed in order to offer the fish trade and consumers simple and practical strategies for the quality retention of this seafood.

Ensuring the quality and safety of seafood has become a paramount concern worldwide for consumers, producers, and regulatory agencies. Temperature control plays a crucial role in preventing enzymatic and microbiological reactions that can lead to spoilage in seafood [7,8]. Among the various preservation techniques employed for seafood, the use of ice is a widely accepted method [9]. Although this method significantly extends the average shelf-life time of fish and shellfish, biochemical and microbial processes can still lead to a noticeable decrease in the sensory quality of chilled fish [10,11]. To address this concern, the industries involved in fish preservation are nowadays exploring more effective natural approaches to maintain the high quality of the product and ensure the consumers’ safety [12,13]. The development of ice with preservative properties has gained remarkable interest in recent years due to its potential to reinforce food safety and promote human health [14,15]. Recent studies have focused on enhancing the storage properties of marine species by employing ice containing different kinds of preservative compounds. These studies have investigated the efficacy of various natural preservatives, such as organic acids (ascorbic, lactic, and citric acids) [16,17] and different kinds of plant extracts like betel leaf (*Piper bettle*) (4-methoxy-isophthalic acid, phenol, and propanoic acid) [18], rosemary (water-soluble compounds) (*Rosmarinus officinalis*) [19], pistachio (*Pistachia vera*) (phenolic compounds) [20], or *Garcinia* sp. (aqueous phenolic compounds) [21].

Macroalgae have attracted great attention as a natural source of compounds with antioxidant, antimicrobial, anti-inflammatory, and anti-cancer properties [7,22,23]. These beneficial properties can be explained on the basis of the presence of preservative metabolites, such as polyphenols, terpenes, phlorotannins, and fucoxanthin. Brown algae, in particular, have proved promising antioxidant activity due to the presence of various polysaccharides including fucoidan, laminarin, and alginic acid [24,25,26]. Among brown algae, the *Cystoseira* genus, belonging to the *Sargassaceae* family, comprises approximately 40 species and is found along the Eastern Atlantic and Mediterranean coastlines [27]. Previous studies have focused on the extraction and purification of bioactive compounds from species belonging to this genus. According to the different kinds of in vitro assays, water-soluble compounds (i.e., fucoidan and sodium alginate compounds) obtained from *Cystoseira compressa* showed remarkable antioxidant properties [28]. A valuable presence of phlorotannin compounds and a notable antioxidant activity (DPPH assay) were detected in crude extracts from *Cystoseira trinodis* collected from the Indian coast [29]. Debromolaurinterol and fucosterol, obtained from different kinds of extracts (petroleum ether, dichloromethane, or methanol) from *Cystoseira myrica* and *C. trinodis*, were found to be responsible for the antioxidant and antimicrobial properties in different in vitro assays [30]. A brassinosteroid-related metabolite with anticarcinogenic properties was extracted and isolated from ethanolic extracts of *C. myrica* [31]. Regarding seafood systems, the presence of an aqueous ethanol extract of *C. compressa* in the icing medium led to an inhibitory effect on the lipid damage and microbial activity development of chilled horse mackerel (*Trachurus trachurus*) [32]. Additionally, the addition of an aqueous extract of *Cystoseira stricta* to the glazing medium led to a remarkable decrease in the lipid oxidation development in frozen Atlantic Chub mackerel (*Scomber colias*) [33].

In agreement with the above-mentioned considerations, the present study addressed the quality enhancement of chilled farmed rainbow trout (*O. mykiss*). For this, ice containing different kinds of *C. myrica* and *C. trinodis* extracts was employed as the icing system during the chilled storage of this fish species. The evolution of the chemical changes and microbial development were determined in the fish muscle throughout a 16-day storage period. The validity of this novel, simple, and practical strategy was verified via a comparison to a fish Control batch stored under traditional ice, i.e., ice prepared only with water.

## 2. Materials and Methods

The chemical reagents and solvents employed were of reagent grade (Merck, Darmstadt, Germany); otherwise, the supplier is expressed.

### 2.1. Extraction of Seaweed

The brown algae *C. myrica* and *C. trinodis* were collected from the coast of Qeshm Island (Iran) in the Persian Gulf and were identified in the Faculty of Science, Guilan University (Rasht, Iran). A voucher specimen was deposited at the herbarium of Guilan University. Once in our laboratory, the algae were thorough washed, dried, and finely powdered in the shade [31].

Both powdered algae were then subjected to extraction using absolute ethanol, distilled water, or a water–ethanol (1:1, *v*/*v*) mixture (15 g dried alga·240 mL^−1^ in all cases), respectively. For this, the suspensions were homogenized (Ultraturrax, IKA, Taufkirchen, Germany) for 30 s, immersed in a magnetic stirrer (Vortex, Scientific Industries, Bohemia, NY, USA) for 12 h, and sonicated (J. P. Selecta, S. A., Barcelona, Spain) for 5 min at 40 KHz (1 s on and 1 s off) in a container including water and ice. Then, the samples were centrifuged (Beckman Coulter ALLEGRA X12R, Brea, CL, USA) at 3500× *g* (10 min, 4 °C), and the resulting supernatants were isolated and subjected to lyophilization (FD8515-C60, Ilshin Biobase Europe, Ede, The Netherlands).

### 2.2. Icing Systems Preparation

To prepare the icing systems, 4.2 mg of each lyophilized extract was mixed with distilled water (2 × 12 mL). The mixture was stirred for 30 s and centrifuged at 3500× *g* for 10 min at 4 °C. Then, the supernatant (20 mL) was taken and diluted to 6 L with distilled water (0.7 mg lyophilized extract·L^−1^ water solution). This solution was stored in a polyethylene bag at −18 °C and employed as the icing system for fish storage. This process was carried out for each kind of extract for both algae. Additionally, traditional or common ice was obtained from distilled water (6 L), packaged, and stored at −18 °C. This ice was employed as the Control condition.

Prior to being used, all icing systems were ground in order to obtain flaked ice. Then, flaked ice was added to the fish in order to carry out the different storage experiments.

### 2.3. Preliminary Trials

The concentration of algal extracts in the icing media used in this study was chosen according to previous preliminary trials. As a first step, the three kinds of lyophilized extracts of both algae were assessed for their antibacterial activities (MIC and MBC assays) on different kinds of bacteria; as a result, increased microbial inhibition (Gram-negative and Gram-positive bacteria) was detected by increasing the extract concentration. Additionally, icing systems containing different concentrations of all kinds of lyophilized extracts (0.1–1 mg·L^−1^ solution range) of both algae were prepared and employed for a storage trial of rainbow trout. During this storage period, possible modification of the natural external color and odor of the fish due to the presence of the algal extract was analyzed. This evaluation aimed to identify the appropriate concentration of the extract that did not compromise the natural color and odor of the fish and maintained a valuable antimicrobial activity. As a result, a concentration of 0.7 mg lyophilized extract·L^−1^ solution showed to be the most concentrated one that did not influence the mentioned sensory descriptors. Consequently, this concentration was considered for carrying out the present study.

### 2.4. Raw Fish, Chilled Storage, and Sampling

A total of 105 rainbow trout (*O. mykiss*) (130 ± 25 g and 22 ± 2 cm) were obtained from a breeding pond (Gorgan, Golestan Province, Iran). The fish were transferred to the laboratory on ice and divided into 7 groups with 15 fish in each. The different fish groups were introduced in separated boxes and directly surrounded by different types of ice. The different batches were as follows: ice without algal extract (Control batch), ice containing aqueous extract from *C. myrica* (MW batch) and *C. trinodis* (TW batch), ice containing ethanolic extract from *C. myrica* (ME batch) and *C. trinodis* (TE batch), and ice containing aqueous–ethanolic extract from *C. myrica* (MWE batch) and *C. trinodis* (TWE batch).

All fish samples were stored in isolated boxes and kept in a refrigerated room (4 ± 1 °C). The fish-to-ice ratio was maintained at 1:1 (*w*/*w*). Throughout the storage period, the water resulting from ice melting was drained and appropriate amounts of new ice were replenished. The analysis of chilled fish was performed after 0, 4, 8, 12, and 16 days of chilled storage.

### 2.5. Determination of Total Phenolic Content (TPC) of Lyophilized Extracts

The TPC of lyophilized extracts from both algae was quantified using the Folin–Ciocalteu colorimetric method [34]. For this, an aliquot of the sample was added to a sodium carbonate solution and to the Folin–Ciocalteu reagent. The mixture was stored in the dark at room temperature for a 30 min period; then, measurement at 720 nm absorbance was carried out. For quantitative purposes, gallic acid was used as a standard to construct the calibration curve. The results were calculated as mg gallic acid·g^−1^ dried algal extract.

### 2.6. Determination of pH and Free Fatty Acid (FFA) Values

The pH value of rainbow trout muscle was determined (390 pH ISE Meter, Beckman Coulter Inc., Fullerton, CA, USA) via dilution of the fish muscle in distilled water in a 1:10 (*w*/*v*) ratio [35].

The content of FFAs was measured following the method proposed by Rukunudin et al. [36]. For this, 5 g of fish fillet was cut into small pieces and homogenized with 30 mL of chloroform (Ultraturrax, IKA, Taufkirchen, Germany). Then, the fillet particles were separated from the fat-containing fraction via centrifugation (5000× *g*, 5 min, 4 °C). Finally, the lipid fraction was titrated with ethanolic potassium hydroxide by employing a 1% phenolphthalein solution as an indicator. The results are expressed as g FFAs·100 g^−1^ lipids.

### 2.7. Measurement of Lipid Oxidation Development

Lipids from rainbow trout muscle were extracted by using the Bligh and Dyer [37] procedure; this method employs a chloroform/methanol (1/1, *v*/*v*) mixture with a solvent–tissue ratio of 4:1 (*v*/*w*). Quantification was carried out in agreement with Herbes and Allen [38]. The lipid content is expressed as g·100 g^−1^ fish muscle.

The peroxide value (PV) was assessed according to previous research [39]. For this, samples of minced fish were mixed with an acetic acid–chloroform (3:2, *v*/*v*) mixture and filtered by employing a Whatman No. 1 filter paper. To the filtered solution, a saturated potassium iodide solution and a starch solution were added. Titration was carried out against a standard solution of sodium thiosulfate. The PV was calculated using the following equation and expressed as peroxide milliequivalents per kg of sample: PV = {(S × N)/W} × 1000, where “S” is the volume of titration (mL), “N” is the normality of the sodium thiosulfate solution, and “W” is the sample weight (g).

The thiobarbituric acid index (TBA-i) was assessed in agreement with Buege and Aust [40]. The method is based on the reaction between a trichloroacetic acid extract of the fish muscle and a thiobarbituric acid (TBA) solution. The content of TBA reactive substances (TBARSs) was calculated by employing a standard curve obtained with 1,1,3,3-tetraethoxy-propane (TEP). This curve was prepared by employing an aqueous solution of TEP (0.24 moles TEP·L^−1^); for this, a range of 0.1 × 10^−8^ to 4 × 10^−8^ moles of malondialdehyde was used. The value of this index was calculated as mg malondialdehyde·kg^−1^ muscle.

### 2.8. Assessment of Microbial Activity

The bacterial content of the chilled rainbow trout muscle was determined following previous methodologies [41,42,43]. Thus, 10 g muscle portions were thoroughly homogenized with 90 mL of a sodium chloride solution. Then, the homogenized suspension was subjected to successive dilutions, and various culture media were used for surface culturing. For total viable bacteria counts, plate count agar (PCA) culture media were used, with the plates being incubated at 30 °C for 48 h. Similarly, PCA culture medium was utilized for psychrophilic bacteria counts, the plates being incubated at 4 °C for 5 to 10 days. To assess *Enterobacteriaceae* counts, Violet Red Bile Agar was used for surface culturing, and the samples were incubated at 37 °C for 24 h. For the identification of bacteria with proteolytic and lipolytic phenotypes, casein–agar and tributyrin–agar media were employed, respectively. The incubations of both media were performed at 30 °C for 48 h. In all bacterial groups, counts were calculated as log CFU·g^−1^ muscle.

### 2.9. Statistical Analysis

Statistical analysis of chemical and microbial values was carried out by employing SAS^®^ software version 9.4. (SAS Campus Drive, Cary, NC, USA). A completely randomized design was employed to analyze the data using a split plot test over time. Subsequently, a mean comparison was performed using the Tukey test at a 95% confidence level. The effect of algal extract in the icing medium was studied. Each processing condition was performed by carrying out three replicates (*n* = 3).

## 3. Results and Discussion

### 3.1. Determination of the TPC of Dried Algal Extracts

**Figure 1:**
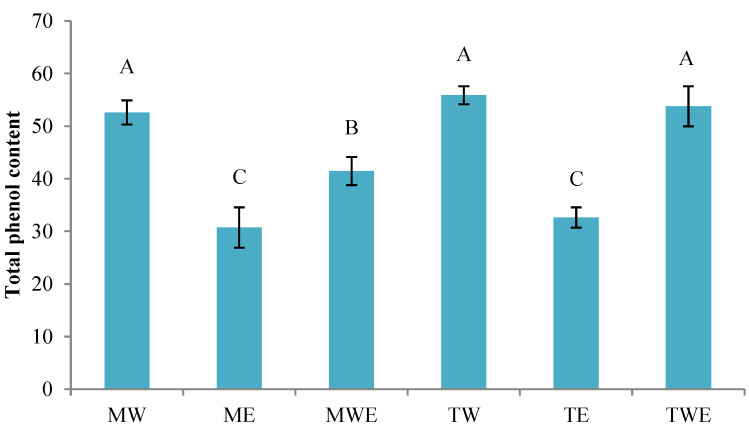
Determination of total phenolic compound contents (mg gallic acid·g^−1^ dried algal extract) in different algal extracts. Mean values of three (*n* = 3) replicates; standard deviations are indicated with bars. Mean values with different letters (A–C) indicate significant (*p* < 0.05) differences. Algal extract abbreviations: MW (water extract of *C. myrica*), ME (ethanolic extract of *C. myrica*), MWE (water–ethanolic extract of *C. myrica*), TW (water extract of *C. trinodis*), TE (ethanolic extract of *C. trinodis*), and TWE (water–ethanolic extract of *C. trinodis*).

The TPCs of crude algal extracts are presented in Figure 1. The range of TPCs was 30.72–55.87 mg gallic acid·g^−1^ dry algal extract. Among the different extracting systems, the highest average contents were found in the aqueous extract (55.87 ± 1.72 mg gallic acid·g^−1^) and in the aqueous–ethanolic extract (53.76 ± 3.83 mg gallic acid·g^−1^) of *C. trinodis*, followed by the aqueous extract (52.60 ± 2.31 mg gallic acid·g^−1^) of *C. myrica*. Such algal extracts showed higher (*p* < 0.05) TPCs than the remaining algal extracts. Notably, ethanol extracts exhibited the lowest (*p* < 0.05) TPCs in both species.

Seaweeds contain a great diversity of water-soluble compounds such as proteins, peptides, sulfated polysaccharides, glycosides, salts, and low-molecular-weight organic acids [24,25,26]. Compared to green and red algae, brown ones exhibit higher levels of phenolic compounds with a remarkable presence of phlorotannin molecules [44,45]. The number of phenolic compounds in algae can fluctuate because of factors such as environmental conditions, season, or habitat [34,46]. Additionally, the choice of the extracting solvent can significantly influence the chemical composition of the extracts, as highlighted by Sathya et al. [29] and Begum et al. [30]. Compared to the present results, higher TPC values (40.8 ± 8.3 mg gallic acid·g^−1^ dry alga) were detected in the brown alga *Bifurcaria bifurcata* [47] but lower (11.0 ± 1.0 mg gallic acid·g^−1^ dried alga) in the brown alga *Undaria pinnatifida* [48].

A remarkable correlation between antioxidant capacity and polyphenol content was proved during the extraction using different solvents (water, ethanol, methanol, and water–methanol, 1:1, *v*/*v*) of the brown alga *Stypocaulon scoparium* [46]; thus, the aqueous extract showed the highest antioxidant activity (DPPH and RSA assays) and the highest phenolic compound content, with gallic acid being the most abundant. The authors indicated that water, being more polar than ethanol and methanol, facilitated the extraction of a greater amount of hydrophilic phenolic compounds [46]. This finding is in agreement with the results obtained in the current study, which indicates that extracts including water (i.e., aqueous and aqueous ethanol extractions) yielded better outcomes in terms of phenolic compounds. Water-soluble compounds (proteins, peptides, sulfated polysaccharides, salts, glycosides, and low-molecular-weight organic acids) have shown to be present in seaweed and be responsible for the preservation properties [49,50]. Notably, aqueous extracts of brown algae have been reported to contain phenolic preservative compounds like chlorogenic acid, vanillic acid, and caffeic acid, which were not observed in ethanol extracts [51]. Furthermore, previous studies have reported that water can lead to higher extraction yields as compared to other extracting systems [26,51,52].

### 3.2. Determination of the pH Value

**Figure 2:**
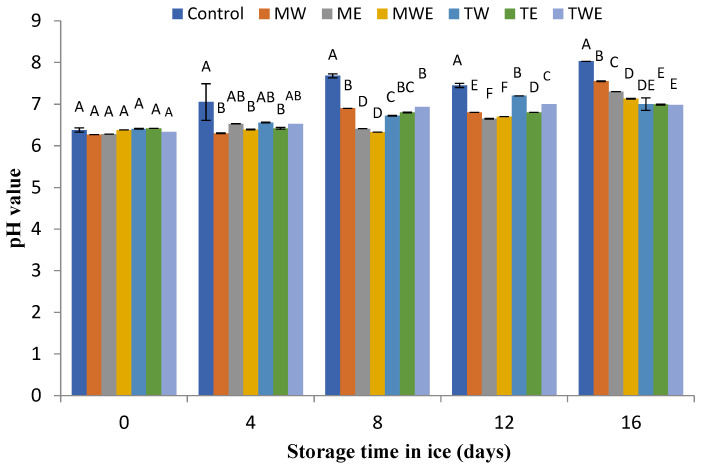
Assessment of the pH value in rainbow trout subjected to different icing systems. Mean values of three (*n* = 3) replicates; standard deviations are indicated with bars. Mean values with different letters (A–E) indicate significant (*p* < 0.05) differences. Abbreviations of icing systems are as expressed in Figure 1.

The pH evolution of rainbow trout muscle stored in different kinds of ice is included in Figure 2. At day 0, pH values were found in the 6.24–6.27 range in all treatments (*p* > 0.05). However, during the storage period, a remarkable increase in all batches was detected. Notably, samples corresponding to the Control condition revealed higher pH values (*p* < 0.05) than the treated fish throughout the 8–16-day period. At the end of storage, batches corresponding to ice with the *C. trinodis* extract exhibited lower pH values (*p* < 0.05) than their counterparts corresponding to *C. myrica*-treated and Control samples.

The pH value plays a decisive role in influencing the activities of microorganisms and enzymes, thereby affecting the shelf-life time [9]. Researchers have attributed the increase in the pH value during the storage period to the activity of internal enzymes within the carcass, as well as to the activity of decay bacteria. These microorganisms produce volatile nitrogen compounds, including ammonia, trimethylamine, and other amine compounds, which subsequently contribute to the pH increase [53,54].

According to the present results, previous research accounts for the inhibition of a pH increase during the chilled storage by including brown algal extracts in the icing medium. This is the case of a combined ethanol–water extract of *C. stricta* during the chilled storage of horse mackerel (*T. trachurus*) for 11 days [32] and an ethanolic extract of *Bifurcaria bifurcata* during the megrim (*Lepidorhombus whiffiagonis*) storage for 14 days [47]. Regarding red algae, Arulkumar et al. [55] observed a pH decrease by including a methanolic extract of *Gracilaria verrucosa* in the icing medium employed during the chilled storage of Indian mackerel (*Rastrelliger kanagurta*) for 15 days.

### 3.3. Assessment of the FFA Content

**Figure 3:**
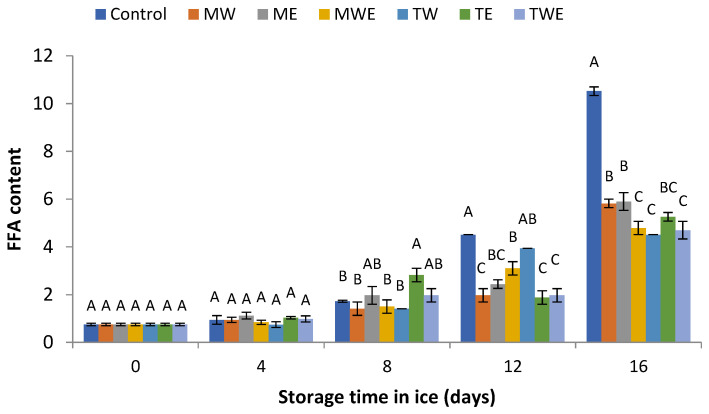
Assessment of the free fatty acid value (g·100 g^−1^ lipids) in rainbow trout subjected to different icing systems. Mean values of three (*n* = 3) replicates; standard deviations are represented with bars. Mean values with different letters (A–C) indicate significant (*p* < 0.05) differences. Icing systems are as expressed in Figure 1.

The data illustrated in Figure 3 show the changes in the FFA content of fish stored in different kinds of ice for 16 days. At day 0, fillets from all kinds of samples were characterized by a ca. 0.75 FFA level. Then, a progressive increase in all samples was observed with the storage time. However, during the 12–16-day period, the FFA content of fish samples corresponding to all alga-treated batches showed lower mean values than their corresponding Control samples; differences were found to be significant (*p* < 0.05) in all cases at the end of the experiment. At this time, the ice treatment with aqueous extracts of *C. trinodis* and aqueous–ethanolic extracts of both algae displayed the lowest average values (4.51, 4.79 and 4.70 g FFAs·100 g^−1^ lipids, respectively).

The current findings suggest the protective properties of extracts obtained from the studied *Cystoseira* algae during the storage period, thus preventing the hydrolytic decay of fatty acids in the fish tissue. This result can be justified by the presence of preservative compounds in the seaweed extracts.

During the chilled storage of fish species, FFA formation has been reported to originate from the activities of fish endogenous enzymes and microorganisms [8,55,56,57]. Before the end of the microbial lag phase is reached, FFAs are signaled to be mostly produced by the activities of endogenous enzymes like phospholipases and lipases [58,59]. Then, an increase of processes related to bacterial catabolism would imply a significant increase in the microbial activity [9,56]. Despite the fact that the formation of FFAs itself does not lead to any notable nutritional loss, the evolution of the FFA content is considered of great interest due to remarkable implications related to quality loss. Thus, increased FFA levels can facilitate the presence of off-tastes and off-odors, detrimental protein changes, and, ultimately, negative effects on the overall quality of the product [9,56,60]. Furthermore, FFA presence has a remarkable effect on lipid oxidation development; thus, FFAs have been reported to present a lower stability to oxidation development than do high-molecular-weight lipid classes (i.e., triacylglycerol and phospholipid molecules) because of reduced steric hindrance to the initial development of oxidative reactions [61,62].

Regarding FFA formation, previous research related to algal species within the present algal genus have been reported for their valuable presence of different kinds of preservative secondary metabolites, such as fatty acids, terpenoids, triacylglycerols, phlorotannins, phenolic compounds, steroids, and polysaccharides [27]. In a seafood system, the addition of a combined ethanol–water extract of *C. stricta* to the icing system employed during horse mackerel (*T. trachurus*) storage for 11 days led to an inhibitory effect on lipid hydrolysis development [32]. Furthermore, the presence of an aqueous extract of the alga *C. stricta* to the glazing medium used during the frozen storage (−18 °C for 9 months) of mackerel (*S. colias*) [33] led to a lower FFA value of fish muscle throughout the frozen storage period.

Regarding brown algae in general, the addition of different kinds of extracts to the icing system used for the chilled storage of marine species has shown to inhibit the evolution of lipid hydrolysis development. This is the case of an ethanolic extract of *B. bifurcata* during a 14-day chilled storage of megrim (*L. whiffiagonis*) [47], a comparative study of aqueous and ethanolic extracts of *B. bifurcata* during a 13-day chilled storage of hake (*Merluccius merluccius*) [63], and an ethanolic extract of *U. pinnatifida* during a 9-day chilled storage of megrim (*L. whiffiagonis*) [48].

### 3.4. Determination of Lipid Oxidation Development

**Figure 4:**
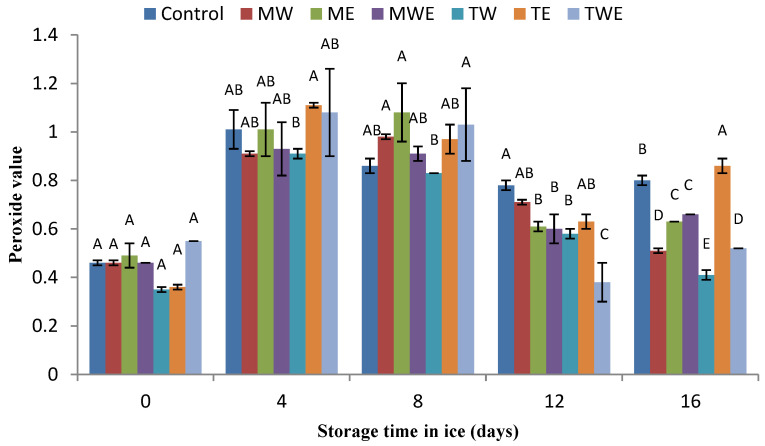
Determination of the peroxide value (meq. active oxygen·kg^−1^ lipids) in rainbow trout subjected to different icing systems. Mean values of three (*n* = 3) replicates; standard deviations are denoted with bars. Mean values with different letters (A–E) indicate significant (*p* < 0.05) differences. Icing systems are as expressed in Figure 1.

**Figure 5:**
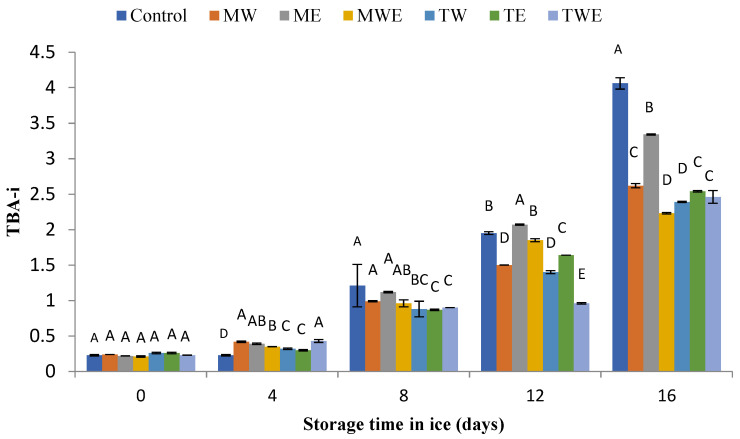
Assessment of the thiobarbituric acid index (TBA-i; mg malondialdehyde·kg^−1^ muscle) in rainbow trout subjected to different icing systems. Average values of three (*n* = 3) replicates; standard deviations are indicated with bars. Average values with different letters (A–E) indicate significant (*p* < 0.05) differences. Icing systems are as expressed in Figure 1.

The evolution of lipid oxidation in chilled fish was determined via the PV (primary oxidation) and the TBA-i (secondary oxidation) (Figure 4 and Figure 5, respectively).

During the ice storage, the PV exhibited an increase after 4 days in all batches (Figure 4). After this time, no subsequent increase was detected in any of the batches under study. Indeed, a general value decrease was detected for the 12–16-day period. Throughout the whole experiment, PVs were, in all cases, below a 1.2 meq·kg^−1^ lipids score, which can be considered as a low level [64]. Some differences among samples corresponding to the different batches could be observed, especially in the 12–16-day period; however, a definite trend about the effect on the PV of the algal extract presence in the icing medium could not be concluded.

The results of the TBA-i are presented in Figure 5. Low values were observed in all batches for the 0–4-day period. Then, a general increase was observed after 8 days of storage, which was followed in all batches by a progressive increase till the end of the experiment. During the 8–16-day period, lower average values were observed in most alga-treated samples than in Control ones; in the case of *C. trinodis*-treated fish, the differences proved to be significant (*p* < 0.05) in all cases. Regarding *C. myrica*-treated samples, batches corresponding to the water extracts showed lower (*p* < 0.05) TBARS values than their corresponding Control samples in the period corresponding to 12–16 days.

The oxidation mechanism of the lipid fraction is described as a multistep process in which various molecules are originated sequentially. Thus, the molecules produced during the initial steps (i.e., primary compounds like peroxides) are relatively unstable and would produce lower-molecular-weight molecules (i.e., secondary compounds like carbonyls) [9,64]. In the subsequent steps of the development of the lipid oxidation mechanism, both kinds of electrophilic compounds (peroxides and carbonyls) would react with compounds including nucleophilic groups like -NH_2_ or -SH (namely proteins, peptides, free amino acids, aminated phospholipids, etc.) in the fish muscle; such reactions would lead to important losses in the fish product regarding acceptability (sensory acceptance decrease) and nutritional value (detrimental changes of the protein fraction) [65,66,67].

In the present research, the general low peroxide level indicates that a remarkable breakdown of peroxides has occurred (Figure 4). However, the presence of preservative compounds in alga-treated fish led to a lower (*p* < 0.05) level of TBARSs than that detected in the Control fish (Figure 5). The relative reduction in oxidation development in treated rainbow trout could be related to polyphenol presence in the extracts, which may contribute to a reduction in oxidation development on the basis of their antioxidant properties. These compounds are electron rich and are prone to enter into efficient electron-donation reactions and produce phenoxyl radical species as intermediates in the presence of oxidizing agents [68,69]. Among polyphenols, phlorotannins from *C. trinodis* have been shown to stabilize phenoxyl radicals through hydrogen bonds with an adjacent hydroxyl group [29].

Previous research indicates that species within the *Cystoseira* genus include a wide range of secondary metabolites, i.e., terpenoids, steroids, phlorotannins, phenolic compounds, and polysaccharides (meroditerpenes and toluquinols), which exhibit potent antioxidant properties [27,70]. Thus, a combined ethanolic-aqueous extract of *C. stricta* was present in the icing system employed for the 11-day chilled storage of horse mackerel (*T. trachurus*) [32]; remarkably, decreased lipid oxidation (fluorescent compound formation) development was detected. Different kinds of extracts (petroleum ether, dichloromethane, or methanol) from *C. myrica* and *C. trinodis* showed a remarkable antioxidant activity (DPPH assay) [30]; this effect was shown to vary according to the extracting solvent employed. Two polysaccharides (i.e., a fucoidan and a sodium alginate) obtained from *C. compressa* showed remarkable antioxidant properties (ferrous ion chelation, ferric ion reduction, and DPPH radical scavenging) [28]. An aqueous extract of the alga *C. stricta* was added to the glazing system used for the frozen (−18 °C for 9 months) storage of Chub mackerel (*S. colias*) [33]; as a result, decreased lipid oxidation (assessment of peroxide, TBA, and fluorescence indices) development and a notable retention of polyunsaturated fatty acid and alpha-tocopherol values was observed.

Previous studies have also shown the antioxidant properties of brown algal extracts in general when employed for the quality retention of chilled marine species. Thus, ethanolic extracts of *B. bifurcata* inhibited the formation of lipid oxidation compounds (i.e., fluorescent compound formation) during the 14-day chilled storage of megrim (*L. whiffiagonis*) [47]. The addition of aqueous, ethanolic, or ethanolic–aqueous extracts of *Fucus spiralis* to the icing media used for the chilled storage of hake (*M. merluccius*) inhibited lipid oxidation development (TBA and fluorescence indices) [71]. An ethanolic extract of *U. pinnatifida* showed a remarkable antioxidant capacity (DPPH assay) and inhibited fluorescent compound formation in chilled megrim (*L. whiffiagonis*) when included in the icing medium during a 9-day storage period [48]. The addition of aqueous or ethanolic extracts of *B. bifurcata* to the icing system used during the 13-day chilled storage of hake (*M. merluccius*) was comparatively studied [63]; neither extract provided a different effect, but both provoked the decreased formation of secondary lipid oxidation molecules (TBARSs) when compared to the Control condition.

### 3.5. Determination of Microbial Activity

**Figure 6:**
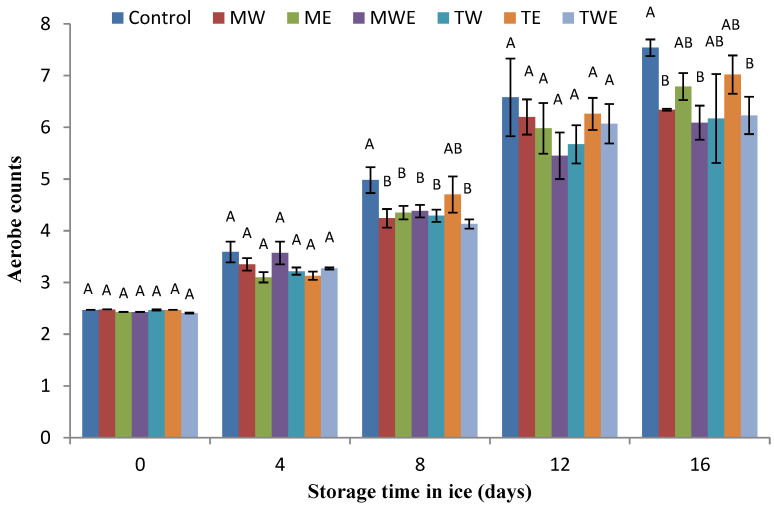
Determination of aerobe counts (log CFU·g^−1^ muscle) in rainbow trout subjected to different icing systems. Average values of three (*n* = 3) replicates; standard deviations are indicated with bars. Average values with different letters (A,B) indicate significant (*p* < 0.05) differences. Icing systems are as expressed in Figure 1.

**Figure 7:**
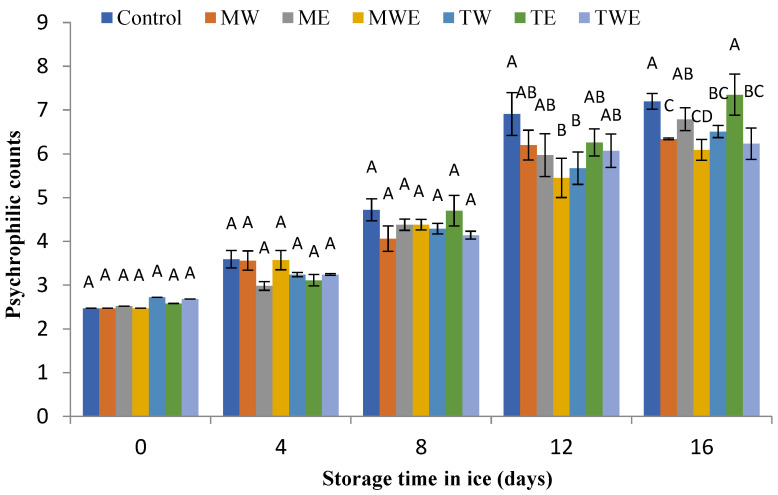
Determination of psychrophilic counts (log CFU·g^−1^ muscle) in rainbow trout subjected to different icing systems. Average values of three (*n* = 3) replicates; standard deviations are indicated with bars. Average values with different letters (A–D) indicate significant (*p* < 0.05) differences. Icing systems are as expressed in Figure 1.

**Figure 8:**
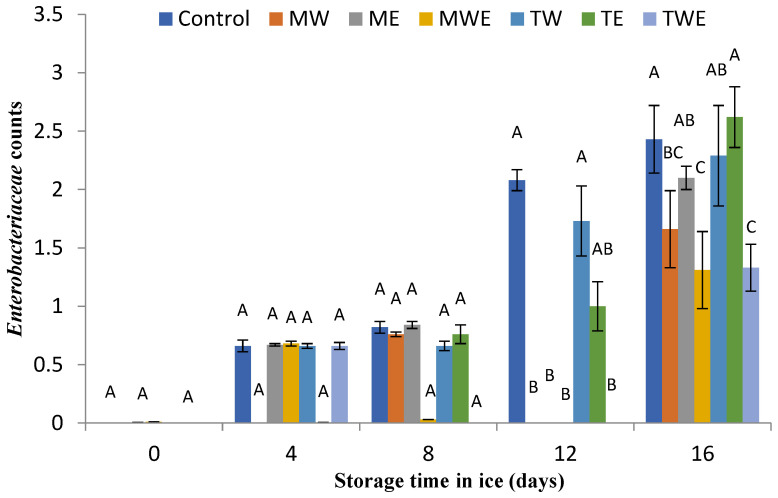
Determination of *Enterobacteriaceae* counts (log CFU·g^−1^ muscle) in rainbow trout subjected to different icing systems. Average values of three (*n* = 3) replicates; standard deviations are indicated with bars. Average values with different letters (A–C) indicate significant (*p* < 0.05) differences. Icing systems are as expressed in Figure 1.

**Figure 9:**
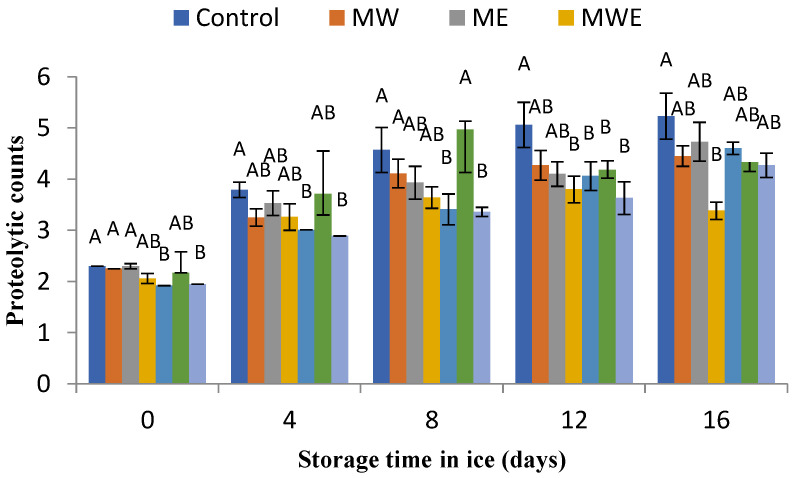
Determination of proteolytic counts (log CFU·g^−1^ muscle) in rainbow trout subjected to different icing systems. Average values of three (*n* = 3) replicates; standard deviations are indicated with bars. Average values with different letters (A,B) indicate significant (*p* < 0.05) differences. Icing systems are as expressed in Figure 1.

**Figure 10:**
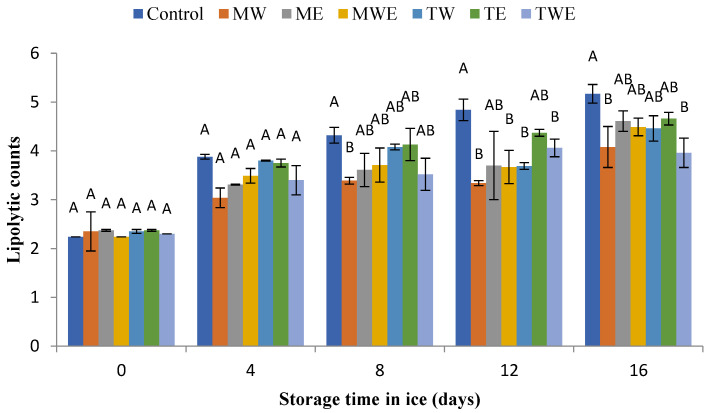
Determination of lipolytic counts (log CFU·g^−1^ muscle) in rainbow trout subjected to different icing systems. Average values of three (*n* = 3) replicates; standard deviations are indicated with bars. Average values with different letters (A,B) indicate significant (*p* < 0.05) differences. Icing systems are as expressed in Figure 1.

The determination of the microbial activity evolution in chilled fish muscle was carried out via the assessment of total aerobe, psychrophilic, *Enterobacteriaceae*, proteolytic, and lipolytic counts (Figure 6, Figure 7, Figure 8, Figure 9 and Figure 10, respectively).

Mesophilic bacterial counting is recognized as a worthwhile tool for evaluating the quality and pollution of fishery products during or after processing [41,42]. In the present study, the initial total bacterial load for rainbow trout muscle ranged from 2.47 to 2.84 CFU·g^−1^ (Figure 6). An overall increase in the total bacterial count across all treatments was detected during storage. No differences (*p* > 0.05) were observed among batches during the 0–4-day period. However, lower average values were detected in all alga-treated fish than in Control samples after 8 days; such differences were found to be significant (*p* < 0.05) in most cases. At day 16, aqueous–ethanolic extracts of both algae led to lower (*p* < 0.05) aerobe counts than in the Control fish.

Psychrophilic bacterial counts play a relevant role in the deterioration of fresh fish and shellfish [72]. Figure 7 displays the changes obtained in psychrophilic bacteria counts for the Control and alga-treated fish during the present 16-day storage in ice. A progressive count increase in all batches was detected with the chilling time, so that a similar growth pattern was observed to that of aerobic bacteria. The initial count of psychrophilic bacteria ranged from 2.47 to 2.58 log CFU·g^−1^. No significant differences (*p* > 0.05) were detected among batches for the 0–8-day period. Then, most alga-treated samples revealed lower average values than those corresponding to the Control condition; the differences were found to be significant (*p* < 0.05) in fish corresponding to the aqueous extract of *C. myrica* (day 16), the aqueous extract of *C. trinodis* (days 12 and 16), the aqueous ethanol extract of *C. myrica* (days 12 and 16), and the aqueous–ethanolic extract of *C. trinodis* (day 16).

During ice storage, psychrophilic bacteria may still maintain certain physiological activities and tolerate cold conditions [41,42]. This activity can be explained on the basis of the development of cold shock proteins, the maintenance of membrane fluidity, and the presence of intracellular enzymes and compatible solutes. It has been shown that common foodborne pathogenic bacteria, such as *Listeria monocytogenes* and *Yersinia enterocolitica*, can survive and grow in cold environments [73]; this fact may signify a potential risk to the microbial safety of ice during fish and shellfish preservation and consumption.

Figure 8 displays the changes in *Enterobacteriaceae* bacteria counts in rainbow trout samples during storage in ice. Up to 8 days of storage, the *Enterobacteriaceae* counts in the Control treatment and groups containing extracts of both algae were not different (*p* > 0.05). However, for the 12–16-day period, the counts in Control fish showed higher average values than most alga-treated samples; the differences were significant (*p* < 0.05) in fish subjected to aqueous extracts of *C. myrica* (days 12 and 16) and in aqueous ethanol extracts of *C. myrica* (days 12 and 16) and *C. trinodis* (days 12 and 16).

In the present study, the count evolution of microorganisms with a proteolytic phenotype is depicted in Figure 9. The results of this bacterial group showed similarities with the above-mentioned aerobe and psychrophilic count evolutions, which showed progressive increases with the storage time. Compared to the Control samples, lower values (*p* < 0.05) were detected in the 0–12-day period in fish corresponding to batches including water and water–ethanol extracts of *C. trinodis*. Additionally, the presence of aqueous–ethanolic extracts of *C. myrica* in the icing system led to lower levels (*p* < 0.05) than in Control samples in the 12–16-day period.

Proteolytic bacteria are specific bacteria that have been signaled to provide unfavorable modifications by contributing to protein breakdown and subsequent textural and nutritional losses of the fish muscle [47,71]. In the current chilled storage, a growing decrease in rainbow trout muscle was concluded for proteolytic bacteria. Therefore, an important preservative effect of the current fish against events related to protein hydrolysis of microbial origin would be implied by the presence of the algal extracts in the icing system.

The study of lipophilic bacteria was also carried out in the present study. This bacteria group is reported to increase lipid hydrolysis development, a mechanism with a notable deteriorative effect on fish quality during chilled storage by facilitating protein denaturation and lipid oxidation events [44]. The results obtained regarding the capacity of lipolytic bacteria to originate extracellular lipases with activity against lipid classes like TAGs and PLs in the current rainbow trout muscle are displayed in Figure 10. In agreement with the above-described results for other bacterial groups, the lipolytic bacteria count increased in all kinds of samples with the storage time. In Control samples, the population of these bacteria increased from 2.24 to 5.17 CFU·g^−1^ by taking into account the initial and end times of the storage period, respectively. However, this lipolytic bacterial development was partially inhibited by the addition of the algal extracts to the icing medium. Thus, an inhibitory effect was detected for the 8–16-day period (water extract of *C. myrica*), 12–16-day period (aqueous–ethanolic extract of *C. trinodis*), and day 12 (aqueous–ethanolic extract of *C. myrica* and aqueous extract of *C. trinodis*).

As a possible explanation, lipolytic bacteria would interfere with the hydrolysis of fats into fatty acids and glycerol by producing lipase enzymes [56]. The present inhibitory effect can be related to the stronger impact of the algal extracts in reducing the activity of lipolytic bacteria regarding fat hydrolysis and further oxidation. Furthermore, algal extracts may quench bacterial enzymatic reactions associated with fat oxidation in fish, according to the above-mentioned results regarding lipid hydrolysis (Figure 3) and oxidation (Figure 5) development.

The decreased microbial activity observed in the current research is in agreement with the polyphenol compound presence in all tested algal extracts. It has been demonstrated that extracts of brown seaweeds contain notable levels of phenolic compounds and the distinctive phlorotannin profile found in this kind of algae, identified for its antimicrobial properties, mainly comprised phloroglucinol, eckol, and dieckol [74]. Polyphenols and their antimicrobial mechanisms are assigned to the alteration of microbial cell permeability, leading to the loss of internal macromolecules and, additionally, to the modification of membrane functions, interruption of cellular integrity, and, ultimately, causing cell death [44,45,75]. However, it is important to remark that the efficiency of extracted algal compounds may differ according to the specific bacteria, the concentration of the compounds used, and the extraction condition. Regarding *Cystoseira* genus, a combined ethanol–water extract of *C. stricta* was present in the icing system employed for the 11-day storage of horse mackerel (*T. trachurus*) [32]; as a result, decreased microbial development (aerobe, psychrotroph, proteolytic, lipolytic, and *Enterobacteriaceae* counts) was detected. Different kinds of extracts (petroleum ether, dichloromethane, or methanol) from *C. myrica* and *C. trinodis* showed a remarkable antimicrobial behavior against different kinds of microorganisms (*Staphylococcus aureus, Streptococcus pyogenes*, *Pseudomonas aeruginosa*, *Escherichia coli, Candida albicans,* and *Cryptococcus neoformans*) [30]; the intensity of this effect was shown to vary according to the extracting solvent employed.

As for the present results, previous studies have already proved the microbial activity inhibition resulting from the use of different kinds of brown algal extracts during the chilled storage of marine species. Thus, ethanolic extracts of *B. bifurcata* led to the inhibited development of aerobes, psychrotrophs, lipolytic bacteria, proteolytic bacteria, and *Enterobacteriaceae* in megrim (*L. whiffiagonis*) during a 14-day chilled storage [47]. In a comparative study of aqueous, ethanolic, and ethanolic–aqueous extracts of *F. spiralis* employed as the icing condition during a 13-day storage of hake (*M. merluccius*) [71], contrary to the present results, the highest inhibitory effects on microbial development (aerobe, psychrotroph, lipolytic bacteria, and proteolytic bacteria counts) were detected in batches including ethanol extracts. A comparative study of aqueous and ethanolic extracts of *B. bifurcata* employed as the icing medium during the storage of hake (*M. merluccius*) for 13 days was carried out [63]; as a result, both batches showed an inhibitory effect on the microbial development (psychrotrophic and lipolytic counts), but no differences were detected between both kinds of extracts. The addition of ethanolic extracts of *U. pinnatifida* to the icing system provoked a decrease in the aerobe, lipolytic, and proteolytic bacteria counts in megrim (*L. whiffiagonis*) stored for 9 days under chilling conditions [48].

Regarding red macroalgae, a remarkable decrease in mesophilic and psychrophilic bacteria counts was proved during a 15-day chilled storage of Indian mackerel (*R. kanagurta*) by including a methanolic extract of the red alga *G. verrucosa* in the icing system employed [55]; the valuable effect was explained after the isolation and identification of bioactive agents (butylated hydroxytoluene, sulfurous acid, 1,2-propanediol, cyclononasiloxane, and tetracosamethylcyclo-dodecasiloxane) present in the algal methanolic extracts.

## 4. Conclusions

A preservative effect of horse mackerel quality was observed with the presence of both algal extracts in the icing media employed during the chilled storage. The inhibitory behavior (*p* < 0.05) on lipid oxidation development and microbial activity was found to be more important at advanced storage times. In general, water and water–ethanol extracts led to higher (*p* < 0.05) preservative effects than their counterpart ethanol extracts. Additionally, higher (*p* < 0.05) total polyphenol values were detected in water and water–ethanol extracts of both algae than in their counterpart extracts obtained only with ethanol. It is assumed that the presence of preservative hydrophilic compounds has been more important than that of lipophilic ones.

According to the results obtained, the presence of aqueous and aqueous ethanol extracts of both algae in the icing system can be considered a valuable method for preserving the quality of farmed rainbow trout during chilled storage. A strategy for the quality enhancement and commercialization of this species under chilling conditions is proposed by employing different kinds of extracts obtained from both *Cystoseira* species. This new, simple, and practical strategy aligns with the nowadays global interest in the search for new tools related to the replacement of synthetic preservative compounds by others obtained from natural sources in order to produce high-quality seafood and food in general.

Further research is envisaged to fully understand the specific benefits and optimal usage of both macroalgal extracts and scale up its employment to other marine species. According to the consumers’ acceptance and nutritional value requirements, complementary studies regarding the effects on the sensory descriptors and changes in the protein fraction of fish muscle resulting in alga-treated fish ought to be developed. Additionally, further research should also include the isolation (HPLC and chromatographic technologies in general) and identification (mass spectrometry) of active molecules (polyphenols and others), which are involved in the inhibitory mechanisms of lipid oxidation and microbial development and are responsible for the preservative behavior detected. To optimize the use of algal extracts, future studies developing an optimized design (namely a response surface methodology study) and taking into account the independent variables of the algal treatment (concentration of the algal extracts and polarity of the extracting system, i.e., water–ethanol concentration ratio) should be conducted. In such cases, the kind of marine species (wild and farmed; fatty and lean) ought to be taken into account.

## Data Availability

The original contributions presented in the study are included in the article; further inquiries can be directed to the corresponding author.
